# Childhood cognitive ability and self-harm and suicide in later life

**DOI:** 10.1016/j.ssmph.2023.101592

**Published:** 2023-12-29

**Authors:** Matthew H. Iveson, Emily L. Ball, Heather C. Whalley, Ian J. Deary, Simon R. Cox, G. David Batty, Ann John, Andrew M. McIntosh

**Affiliations:** a Centre for Clinical Brain Sciences, The University of Edinburgh, UK; b Department of Psychology, The University of Edinburgh, UK; c Institute of Epidemiology and Health, University College London, London, UK; d Swansea University Medical School, Swansea University, Swansea, UK

**Keywords:** Cognitive ability, Self-harm, Suicide, Older age, Data linkage, Epidemiology

## Abstract

**Background:**

Self-harm and suicide remain prevalent in later life. For younger adults, higher early-life cognitive ability appears to predict lower self-harm and suicide risk. Comparatively little is known about these associations among middle-aged and older adults.

**Methods:**

This study examined the association between childhood (age 11) cognitive ability and self-harm and suicide risk among a Scotland-wide cohort (N = 53037), using hospital admission and mortality records to follow individuals from age 34 to 85. Multistate models examined the association between childhood cognitive ability and transitions between unaffected, self-harm, and then suicide or non-suicide death.

**Results:**

After adjusting for childhood and adulthood socioeconomic conditions, higher childhood cognitive ability was significantly associated with reduced risk of self-harm among both males (451 events; HR = 0.90, 95% CI [0.82, 0.99]) and females (516 events; HR = 0.89, 95% CI [0.81, 0.98]). Childhood cognitive ability was not significantly associated with suicide risk among those with (Male: 16 events, HR = 1.05, 95% CI [0.61, 1.80]; Female: 13 events, HR = 1.08, 95% CI [0.55, 2.15]) or without self-harm events (Male: 118 events, HR = 1.17, 95% CI [0.84, 1.63]; Female: 31 events, HR = 1.30, 95% CI [0.70, 2.41]).

**Limitations:**

The study only includes self-harm events that result in a hospital admission and does not account for self-harm prior to follow-up.

**Conclusions:**

This extends work on cognitive ability and mental health, demonstrating that these associations can span the life course and into middle and older age.

## Highlights

• Examines associations with cognitive ability measured in childhood (age 11 years).
• Uses a large nationwide sample of older adults born around 1936 (N = 53037).
• Uses electronic health records to trace self-harm and suicide from age 34 to 85.
• Self-harm and suicide were relatively rare among older adults.
• Higher age-11 cognition was associated with lower risk of self-harm but not suicide.

## 1 Introduction

Self-harm (i.e., intentional self-injury or poisoning) and suicide are common among middle-aged and older adults. A recent meta-analysis reported yearly rates of between 19 and 65 per 100,000 in older adults (aged 60+) for self-harm (Troya et al., 2019) and recent UK estimates show suicide death rates peak in middle-age (England and Wales: 15 per 100,000; Scotland: 21 per 100,000) and drop only slightly (England & Wales: 10 per 100,000; Scotland: 12 per 100,000) after the age of 60 (ONS, 2021; Scottish Public Health Observatory, 2022b).

A number of psychosocial risk factors have been identified for self-harm and suicide, including socioeconomic disadvantage, social isolation, chronic distress and psychiatric disorders (Batty et al., 2018), family size (Riordan et al., 2012) and family position (Grinde & Tambs, 2016). Among such risk factors, cognitive ability (intelligence) has been associated with both self-harm and suicide risk. Evidence from large cohort studies suggests that higher cognitive ability is associated with lower risk of self-harm (Batty et al., 2010; Fergusson et al., 2005; Jiang et al., 1999; Osler et al., 2008) and lower risk of suicide (Allebeck et al., 1988, p. 988; Andersson et al., 2008; Christensen et al., 2016; Gunnell et al., 2005; Hemmingsson et al., 2006; Osler et al., 2008; O'Toole & Cantor, 1995). However, the majority of these studies focus on self-harm and suicide risk up to middle-age, particularly among males. The epidemiology of self-harm and suicide may change in later life, and it is unclear whether cognitive ability also predicts self-harm and suicide risk into middle and older age, for both males and females. Furthermore, the majority of studies use cognitive ability measured in early-adulthood; using a measure from childhood may minimise the impact of confounders such as education and undetected psychiatric conditions (Hatch et al., 2007; Kuh et al., 2004).

Only one study has examined the association between childhood cognitive ability and suicide into later-life for both males and females. In a nationwide sample of Scottish adults up to the age of 79, Calvin et al. (2017) reported that higher childhood cognitive ability was associated with a reduced risk of suicide among males (HR = 0.80, 95% CI [0.66, 0.96]), but not among females (HR = 1.15, 95% CI [0.82, 1.60]). No studies to date have examined the impact of childhood cognitive ability on self-harm in middle-aged and older adults. Although self-harm and suicide share common risk factors (Duarte et al., 2020), it is unclear whether childhood cognitive ability is a risk factor for suicide specifically, or whether its contribution may be mediated by self-harm.

The majority of work – including that in later-life (Calvin et al., 2017) – does not consider both self-harm and suicide concurrently. This makes it difficult to estimate the independent contribution of cognitive ability. Although self-harm and suicide are distinct outcomes, self-harm is one of the most important risk factors for suicide, present in almost half of those who go on to take their own lives (Chan et al., 2016; Hawton et al., 2003). Indeed, the risk of suicide among older adults presenting to hospital with self-harm has been shown to be 67 times higher than the general population of older adults (Murphy et al., 2012). Self-harm may act as a mediator of the association between cognitive ability and suicide risk. Examining self-harm and suicide concurrently is important for disentangling risk, and for estimating the association between childhood cognitive ability and suicide separately among those with and without self-harm behaviours.

The present study examines the association between childhood cognitive ability and later-life self-harm and suicide in a population birth cohort recruited in childhood. After follow-up for suicide risk in the Scottish Mental Survey 1947 cohort (Calvin et al., 2017), the present study uses multistate models to examine the independent, concurrent associations between childhood cognitive ability and transitions into both self-harm and suicide. The present study also extends the follow-up period to cover routinely-collected health records from middle-age up to age 85.

## 2 Methods

### 2.1 Sample

A flow diagram detailing sample selection is shown in Supplementary Material (Supplementary Fig. 1). The initial sample was formed of 70,805 individuals who took part in the Scottish Mental Survey 1947 (SMS1947) (The Scottish Council for Research in TheScottish Council for Research in Education, 1949), a nationwide assessment of cognitive ability and socioeconomic conditions administered to 1936-born individuals attending a school in Scotland in June 1947. Around 94% of eligible children took part in the survey, with the remainder primarily being absent on the day of testing (The Scottish Council for Research in TheScottish Council for Research in Education, 1949). The analytic sample was formed of 53,037 of these individuals who were traceable in Community Health Index (CHI) records. A CHI record is generated upon an individual's first contact with the National Health Service (NHS) in Scotland; individuals are assigned a unique number that is present on most electronic health records enabling them to be linked together. Excluded participants (N = 19,104) either could not be traced in CHI-linked records or had multiple potential matches without a means to reconcile them. Individuals who could not be traced may have left Scotland or died before interacting with the NHS and being assigned a CHI number to enable linkage. Previous work in the same cohort suggests that the majority of non-traced individuals likely died prior to receiving a CHI number (Calvin et al., 2017). De-identified datasets containing person-level data were created by Public Health Scotland Records and were made accessible to named researchers within a secure Trusted Research Environment.

### 2.2 Self-harm-related hospitalisation, suicide and other deaths

Self-harm and suicide outcomes were extracted from routinely-collected national health records. Self-harm-related hospitalisations were taken from Scottish Morbidity Records (SMR), covering all inpatient admissions to a Scottish NHS hospital from January 1980 to March 2021 (age 44 to age 85 years-old). General/acute inpatient and day case (i.e., those admitted but discharged on the same day) (SMR01) and mental health inpatient and day case (SMR04) admissions were examined for ICD9 and ICD10 codes related to intentional self-injury and poisonings (Supplementary Material). Note that this definition does not distinguish between attempted suicide and other reasons for self-harm. However, attempted suicide makes up a greater proportion of self-harm presentations to hospital, particularly among older adults (Draper, 1996; Hawton & Harriss, 2008). The definition of self-harm events in the present sample, then, may predominantly represent suicide attempts. A binary variable was created to indicate whether an individual had experienced a self-harm-related hospital admission and the age (in months) at which the first self-harm admission occurred.

Deaths due to suicide and other causes of death were taken from the National Records of Scotland Death Register, covering all deaths registered in Scotland from January 1970 to March 2021 (age 34 to age 85 years-old). Deaths due to intentional self-injury and poisonings are flagged within the register by the data provider using a list of ICD10 codes (X60-X84) and their ICD9 equivalents. Note that many previous studies – including population estimates by the ONS (ONS, 2021) – use a broader classification that includes deaths due to undetermined events. In Scotland, these are operationalised as ‘probable self-harm’ and are not included in the present definition of suicide. However, self-injury and undetermined (open) verdicts have been shown to be similar in terms of their demographic and medical parameters (Linsley et al., 2001). The flag in death records was used to create a binary variable indicating whether a death had been due to suicide or due to other causes. Age (in months) at which the death occurred was also recorded.

### 2.3 Childhood cognitive ability

Cognitive ability in childhood was measured in school using the Moray House Test No. 12 (MHT), completed by children once on the June 4, 1947, around age 11, as part of the SMS1947 (The Scottish Council for Research in TheScottish Council for Research in Education, 1949). The MHT is a group-administered assessment of general cognitive ability, including items on verbal reasoning, arithmetic and spatial problem solving. Performance on the test was used to inform school selection. More information about the MHT, its context and its administration can be found elsewhere (The Scottish Council for Research in TheScottish Council for Research in Education, 1949). Participants could score a maximum of 76. Raw scores were age-residualised to account for small variations in age at test, before being IQ-scaled (M = 100, SD = 15) and standardised (M = 0, SD = 1) to aid interpretation.

### 2.4 Covariates

The analyses included covariates chosen due to their association with self-harm and suicide in previous work, or due to their role as potential confounders of childhood cognitive ability. Sex (male or female) was taken from routinely-collected NHS CHI records. Childhood family conditions were included as a close correlate of socioeconomic conditions in childhood. Childhood family conditions were measured using a two-item survey completed as part of the SMS1947. For each child participating in the SMS1947, trained teachers were asked to report the number of children present in their family in 1947 (family size) and their position among children in the family ordered by age (family position). Higher numbers in family size indicated larger families, and higher numbers in family position indicated younger age relative to most of their siblings at the time of assessment. Family size was Yeo-Johnson scaled to account for a non-normal distribution. This involves rescaling the variable (a monotonic transformation using power functions similar to a Box-Cox transformation) to result in a distribution that is easier for analytic models to account for.

Socioeconomic deprivation in adulthood were measured by the decile on the Carstairs Index 1991, an area-based measure of relative material deprivation that aggregates male unemployment, low occupational social class, non-car ownership and household overcrowding over a data zone (Carstairs & Morris, 1991; McLoone, 2004). Carstairs deciles are recorded routinely as part of an inpatient or day case admission to secondary care NHS services (e.g., a hospital) in Scotland. For those experiencing self-harm, deciles were taken from the hospital inpatient record associated with a first self-harm admission. For those not experiencing self-harm, deciles were taken from their last recorded inpatient admission (i.e., closest to death, suicide or censoring at end of follow-up). Those without any inpatient admissions were treated as having missing Carstairs 1991 deciles.

### 2.5 Statistical analyses

**Fig. 1:**
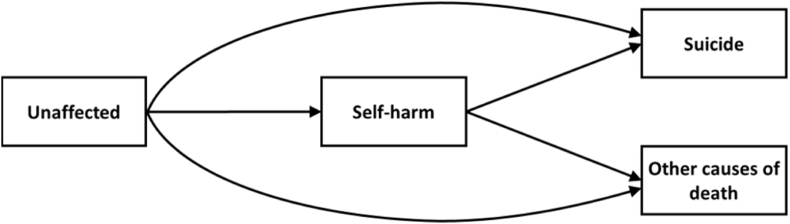
Transition diagram indicating five possible transitions modelled in the analyses. All individuals enter the study in the unaffected state.

Analyses were conducted for males and females separately due to reported sex differences in both self-harm and suicide (Chan et al., 2007; Murphy et al., 2012) and mortality (Nathanson, 1984; Read & Gorman, 2010) risk. Multistate models were used to account for the fact that individuals could move between states of health (unaffected at baseline), self-harm, and suicide and other causes of death in several ways. Fig. 1 models the possible transitions. All individuals entered the study as unaffected, though this does not account for health (including previous episodes of self-harm) prior to entry. Self-harm, suicide and other causes of death were treated as absorbing states, with no return to the unaffected state allowed. Furthermore, suicide and other causes of death were treated as competing states; individuals could only enter one of these states with no transition between them. Time to transition was estimated in months from birth. Individuals were right censored at 1027 months (around 86 years-old) at the end of the follow-up period.

A univariate model was constructed to assess the association between childhood cognitive ability and the hazards of each of the 5 transitions (Fig. 1) simultaneously. Similar univariate models were created for each of the covariates and their associations with the 5 transitions. A multivariate model was then conducted including childhood cognitive ability and covariates (sex, family size, family position, Carstairs decile at admission), as well as time (in months) spent in the self-harm state (i.e., time from a self-harm admission to entry to another state). Note that time in the self-harm state associations were only estimated with transitions from self-harm to suicide or to other death. Missing values in childhood cognitive ability, family size, family position and Carstairs decile were multiply imputed. None of the included variables violated the proportional hazards assumption when visually inspecting the Kaplan-Meier curves or when testing Schoenfeld Residuals against transformed time (all ps > 0.05). This indicates that, for each variable and outcome, the hazard rate of an individual remained relatively constant over time. Given that the follow-up extended across middle- and older-age, we conducted sensitivity analyses using only events that occurred at age 65 years or older to specifically examine associations beyond middle-age (Supplementary Tables S2 and S3). Notably, a very similar pattern of associations was observed, albeit with much wider confidence intervals due to a smaller number of events. Furthermore, the fully-adjusted models were repeated without the adulthood deprivation measure in order to account for potential collider bias from including post-treatment controls (Elwert & Winship, 2014) (Supplementary Tables S4 and S5). The results were very similar to those of the fully-adjusted models that included adulthood deprivation, particularly in regards to the risk of transitions into self-harm and into suicide states.

Analyses were conducted with R (version 4.0.5) (R Core Team, 2021) using the ‘mstate’ package (version 0.3.2) (de Wreede et al., 2011). Multiple imputation was performed using the ‘mice’ package (version 3.16.0) (Buuren & Groothuis-Oudshoorn, 2011).

## 3 Results

In terms of sample selection effects, individuals excluded due to missing CHI records did not significantly differ from those retained in the analytic sample in age (in months) at SMS1947 (excluded: M = 130.75, SD = 3.44; analytic sample: M = 130.75, SD = 3.44; t = −0.18, df = 33823, p = 0.85). However, excluded participants had significantly higher cognitive ability (verbal reasoning, arithmetic and spatial problem solving) in terms of MHT scores (excluded: M = 38.47, SD = 15.76; analytic sample: M = 36.39, SD = 15.71; t = 15.12, df = 31460, p < 0.001), significantly earlier position in family (excluded: M = 2.45, SD = 1.77; analytic sample: M = 2.53, SD = 1.82; t = −5.11, df = 34344, p < 0.001) and significantly smaller families (excluded: M = 3.70, SD = 2.16; analytic sample: M = 3.80, SD = 2.22; t = −5.46, df = 34328, p < 0.001) at age 11.

Descriptive statistics for the analytic sample are shown in Table 1. Of the 53,037 individuals in the analytic sample, 28,097 appeared in death records (N = 178 with a ‘suicide’ cause of death) and 16,895 appeared in hospital inpatient records (N = 967 with a self-harm-related admission). There was no significant difference in the proportion of individuals experiencing self-harm between males and females (X^2^ = 3.14, p = 0.08). However, there were significant demographic differences to justify splitting further analyses by sex: compared to males, females exhibited significantly higher MHT scores at age 11 (mean difference = 2.02, p < 0.001), significantly older age at admission (mean difference = 33.05 months, p < 0.001) and significantly older age at censor (mean difference = 26.59 months, p < 0.001). Additionally, a greater proportion of females were alive at the end of follow-up relative to males (X^2^ = 1024.53, p < 0.001).

**Table 1:** Descriptive statistics for the analytic sample according to sex.

		Female N = 26803	Male N = 26234	Total N = 53037
Age 11 MHT score				
	Mean (SD)	37.39	35.37	36.39
		(14.98)	(16.36)	(15.71)
	N Missing (%)	1773	1753	3526
		(7)	(7)	(7)
**Age 11 position in family**				
	Mean (SD)	2.55	2.51	2.53
		(1.85)	(1.80)	(1.82)
	N Missing (%)	269	226	495
		(1)	(1)	(1)
**Age 11 size of family**				
	Mean (SD)	3.83	3.78	3.80
		(2.25)	(2.20)	(2.22)
	N Missing (%)	272	227	499
**Carstairs decile at hospital admission**				
	Median (IQR)	6	6	6
		(5)	(5)	(5)
	N Missing (%)	17789	18242	36031
		(66)	(70)	(68)
**Age at hospital admission (months)**				
	Mean (SD)	72.09	69.34	70.80
		(10.54)	(11.17)	(10.93)
	N Missing (%)	17729	18175	35904
		(66)	(69)	(68)
**Age at censor (months)**				
	Mean (SD)	77.47	75.26	76.38
		(9.94)	(10.72)	(10.39)
	N Missing (%)	0	0	0
		(0)	(0)	(0)
**Self-harm hospital admission status**				
	N No self-harm (%)	26287	25783	52070
		(98)	(98)	(98)
	N Self-harm (%)	516	451	967
		(2)	(2)	(2)
	N Missing (%)	0	0	0
		(0)	(0)	(0)
**Deceased status**				
	N Alive (%)	14419	10521	24940
		(54)	(40)	(47)
	N Suicide (%)	44 (<1)	134 (<1)	178 (<1)
	N Other Deceased (%)	12340	15579	27919
		(46)	(60)	(53)
	N Missing (%)	0	0	0
		(0)	(0)	(0)

MHT = Moray House Test measuring cognitive ability (out of 76); Carstairs = Carstairs Index 1991 measuring area-level material deprivation (between 1 and 10, with higher deciles indicating greater deprivation).

Transitions between states for males and females are shown in Table 2. Kaplan-Meier curves showing cumulative hazards for each transition are shown in Supplementary Material. The risk of all transitions generally increased with age (Supplementary Fig. 2). Transition risk from unaffected to self-harm was greatest between age 70 and 80. The risk of other transitions was highest after the age of 80. Transitions to non-suicide death were more frequent among males (Cumulative Incidence = 59.38%) than females (Cumulative Incidence = 46.04%). In contrast, no transition (i.e., no self-harm, suicide or death) was more frequent among females (Cumulative Incidence = 53.23%) than males (Cumulative Incidence = 39.78%). Transitions to self-harm were slightly more frequent among females (Cumulative Incidence = 1.93%) than males (Cumulative Incidence = 1.72%). Examining the relative risk of suicide among those with and without self-harm events (hospital admissions) demonstrated a much higher ratio among females (RR = 21.00, 95%CI [11.47, 41.38]) than among males (RR = 7.89, 95%CI [4.72, 13.18]).

**Table 2:** Frequency of each transition, males and females. Diagonals represent those who stayed in each state until the end of follow-up.

	Males					Females				
	N = 26234					N = 26803				
To										
**From**		Unaffected	Self-harm	Suicide	Death		Unaffected	Self-harm	Suicide	Death
	Unaffected	10435	451	118	15230	Unaffected	14266	516	31	11990
	Self-harm	–	86	16	349	Self-harm	–	153	13	350
	Suicide	–	–	134	–	Suicide	–	–	44	–
	Death	–	–	–	15579	Death	–	–	–	12340

### 3.1 Self-harm risk

The univariate and mutually-adjusted associations between each variable and the risk of each transition are shown in Table 3 (Males) and Table 4 (Females). Lower childhood cognitive ability was significantly associated with an increased risk of transitioning from unaffected to self-harm states for males and females, both in the univariate and mutually-adjusted models. In males, a 1SD advantage in childhood cognitive ability childhood cognitive ability was associated with a 10% lower risk of self-harm (HR = 0.90, 95% CI [0.82, 0.99]). A similar strength of association was observed among females (HR = 0.89, 95% CI [0.81, 0.98]). Higher adult material deprivation was significantly associated with increased risk of self-harm transitions among both males and females, including in the mutually-adjusted model. A single decile increase on the Carstairs index of deprivation (indicating more deprivation) was associated with a 7% higher risk of transitioning to self-harm among males (HR = 1.07, 95% CI [1.03, 1.11]) and a 12% increase in risk among females (HR = 1.12, 95% CI [1.08, 1.16]).

**Table 3:** Univariate and mutually-adjusted associations with each transition from the multistate model, males.

**Transition** (N transitioning/N in state)	**Variable** (unit change)	Univariate			Mutually-adjusted		
		**HR**	**SE**	**p-value**	**HR**	**SE**	**p-value**
		**[95% CI]**			**[95% CI]**		
**Unaffected > Self-harm** (N = 451/26234)	**Childhood Cognitive ability**	0.76	0.05	<0.001	0.90	0.05	0.03
	(1SD higher)	[0.70, 0.83]			[0.82, 0.99]		
	**Childhood Family position**	1.11	0.05	0.03	1.03	0.07	0.72
	(1 person later)	[1.01, 1.21]			[0.89, 1.18]		
	**Childhood Family size** (1 person larger)	1.06 [1.02, 1.10]	0.02	0.01	0.99 [0.93, 1.05]	0.03	0.66
	**Adulthood Carstairs deprivation** (1 decile more deprived)	1.08 [1.05, 1.12]	0.02	<0.001	1.07 [1.03, 1.11]	0.02	<0.001
**Unaffected > Suicide** (N = 118/26234)	**Childhood Cognitive ability** (1SD higher)	0.84	0.09	0.05	1.17 [0.84, 1.63]	0.17	0.35
		[0.70, 1.00]					
	**Childhood Family position** (1 person later)	1.15 [0.96, 1.38]	0.09	0.12	1.07 [0.68, 1.69]	0.23	0.68
	**Childhood Family size** (1 person larger)	1.06 [0.99, 1.15]	0.04	0.11	1.03 [0.85, 1.26]	0.10	0.75
	**Adulthood Carstairs deprivation** (1 decile more deprived)	1.07 [0.96, 1.20]	0.06	0.22	1.09 [0.97, 1.22]	0.06	0.17
**Unaffected > Non-suicide death** (N = 15230/26234)	**Childhood Cognitive ability** (1SD higher)	0.84 [0.83, 0.86]	0.01	<0.001	0.95 [0.92, 0.98]	0.01	<0.001
	**Childhood Family position** (1 person later)	1.10 [1.08, 1.12]	0.01	<0.001	1.04 [1.00, 1.08]	0.02	0.06
	**Childhood Family size** (1 person larger)	1.04 [1.04. 1.05]	0.01	<0.001	1.00 [0.99, 1.02]	0.01	0.61
	**Adulthood Carstairs deprivation** (1 decile more deprived)	1.06 [1.05, 1.07]	0.01	<0.001	1.06 [1.05, 1.07]	0.01	<0.001
**Self-harm > Suicide** (N = 16/451)	**Childhood Cognitive ability** (1SD higher)	1.22 [0.73, 2.07]	0.27	0.45	1.05 [0.61, 1.80]	0.28	0.86
	**Childhood Family position** (1 person later)	1.17 [0.71, 1.94]	0.26	0.54	2.91 [1.14, 7.43]	0.48	0.03
	**Childhood Family size** (1 person larger)	0.94 [0.75, 1.18]	0.12	0.59	0.64 [0.40, 1.01]	0.23	0.06
	**Adulthood Carstairs deprivation** (1 decile more deprived)	0.87 [0.73, 1.03]	0.09	0.10	0.90 [0.75, 1.08]	0.09	0.26
	**Time in self-harm state** (1 month longer)	–	–	–	1.01 [1.00, 1.02]	0.01	0.02
**Self-harm > Non-suicide death** (N = 349/451)	**Childhood Cognitive ability** (1SD higher)	0.88 [0.79, 0.98]	0.06	0.02	0.90 [0.80, 1.02]	0.06	0.09
	**Childhood Family position** (1 person later)	0.97 [0.87, 1.08]	0.06	0.56	0.91 [0.78, 1.07]	0.08	0.25
	**Childhood Family size** (1 person larger)	1.03 [0.98, 1.07]	0.02	0.27	1.04 [0.97, 1.12]	0.04	0.24
	**Adulthood Carstairs deprivation** (1 decile more deprived)	1.05 [1.01, 1.09]	0.02	0.02	1.03 [0.99, 1.08]	0.02	0.14
	**Time in self-harm state** (1 month longer)	–	–	–	1.00 [1.00, 1.00]	<0.01	0.16

**Table 4:** Univariate and mutually-adjusted associations with each transition from the multistate model, females.

**Transition** (N transitioning/N in state)	**Variable** (unit change)	Univariate			Mutually-adjusted		
		**HR [95% CI]**	**SE**	**p-value**	**HR [95% CI]**	**SE**	**p-value**
**Unaffected > Self-harm** (N = 516/26803)	**Childhood Cognitive ability** (1SD higher)	0.74 [0.68, 0.81]	0.05	<0.001	0.89 [0.81, 0.98]	0.05	0.02
	**Childhood Family position** (1 person later)	1.13 [1.03, 1.23]	0.04	0.01	1.02 [0.90, 1.16]	0.07	0.72
	**Childhood Family size** (1 person larger)	1.06 [1.02, 1.10]	0.02	<0.001	1.00 [0.95, 1.06]	0.03	0.98
	**Adulthood Carstairs deprivation** (1 decile more deprived)	1.13 [1.10, 1.17]	0.02	<0.001	1.12 [1.08, 1.16]	0.02	<0.001
**Unaffected > Suicide** (N = 31/26803)	**Childhood Cognitive ability** (1SD higher)	1.40 [0.92, 2.13]	0.22	0.12	1.30 [0.70, 2.41]	0.31	0.40
	**Childhood Family position** (1 person later)	1.03 [0.73, 1.46]	0.18	0.86	1.47 [0.65, 3.33]	0.42	0.36
	**Childhood Family size** (1 person larger)	0.95 [0.80, 1.12]	0.09	0.52	0.79 [0.52, 1.19]	0.21	0.26
	**Adulthood Carstairs deprivation** (1 decile more deprived)	1.02 [0.85, 1.21]	0.09	0.86	1.10 [0.91, 1.34]	0.10	0.33
**Unaffected > Non-suicide death** (N = 11990/26803)	**Childhood Cognitive ability** (1SD higher)	0.79 [0.77, 0.80]	0.01	<0.001	0.84 [0.81, 0.87]	0.02	<0.001
	**Childhood Family position** (1 person later)	1.08 [1.06, 1.10]	0.01	<0.001	0.97 [0.93, 1.02]	0.02	0.23
	**Childhood Family size** (1 person larger)	1.04 [1.03, 1.05]	<0.01	<0.001	1.02 [1.00, 1.04]	0.01	0.03
	**Adulthood Carstairs deprivation** (1 decile more deprived)	1.06 [1.05, 1.07]	0.01	<0.001	1.04 [1.03, 1.06]	0.01	<0.001
**Self-harm > Suicide** (N = 13/516)	**Childhood Cognitive ability** (1SD higher)	1.12 [0.64, 1.98]	0.29	0.69	1.08 [0.55, 2.15]	0.35	0.82
	**Childhood Family position** (1 person later)	0.94 [0.54, 1.63]	0.28	0.83	0.65 [0.30, 1.42]	0.40	0.28
	**Childhood Family size** (1 person larger)	1.08 [0.86, 1.36]	0.12	0.51	1.31 [0.92, 1.88]	0.18	0.14
	**Adulthood Carstairs deprivation** (1 decile more deprived)	0.82 [0.68, 0.99]	0.10	0.04	0.87 [0.71, 1.07]	0.11	0.18
	**Time in self-harm state** (1 month longer)	–	–	–	1.04 [1.02, 1.06]	0.01	<0.001
**Self-harm > Non-suicide death** (N = 350/516)	**Childhood Cognitive ability** (1SD higher)	0.86 [0.77, 0.96]	0.06	0.01	0.88 [0.78, 1.00]	0.06	0.04
	**Childhood Family position** (1 person later)	1.07 [0.96, 1.20]	0.05	0.19	1.01 [0.85, 1.19]	0.08	0.93
	**Childhood Family size** (1 person larger)	1.03 [0.99, 1.08]	0.02	0.16	1.01 [0.94, 1.09]	0.04	0.80
	**Adulthood Carstairs deprivation** (1 decile more deprived)	1.04 [1.00, 1.08]	0.02	0.05	1.05 [1.00, 1.10]	0.02	0.03
	**Time in self-harm state** (1 month longer)	–	–	–	1.00 [1.00, 1.01]	<0.01	<0.001

### 3.2 Suicide risk

Among both males and females, the risk of transitioning from unaffected to suicide was not significantly associated with childhood cognitive ability or other covariates in either the univariate or mutually-adjusted models.

Risk of transition from self-harm to suicide was not significantly associated with childhood cognitive ability in either the univariate or mutually-adjusted model, among either males or females. In the mutually-adjusted model, time spent in the self-harm state (before transition or censor) was significantly associated with the risk of suicide, with a single month increase in time associated with a 1% increase in suicide risk among males (HR = 1.01, 95% CI [1.00, 1.02]) and a 4% higher risk of suicide among females (HR = 1.04, 95% CI [1.02, 1.06]). For males only, an increased risk of suicide was also significantly associated with being born later in the family in the mutually-adjusted model. A 1-later birth position (e.g., second child vs. first child) was associated with a 191% higher risk (HR = 2.91, 95% CI [1.14, 7.43]).

### 3.3 Non-suicide death risk

As in previous work in the same cohort (Calvin et al., 2017), an increased risk of transitioning from unaffected to non-suicide deaths was significantly associated with lower childhood cognitive ability, both in the univariate and mutually-adjusted models and among males and females. In the mutually-adjusted model, a 1SD advantage in childhood cognitive ability was associated with a 5% lower risk of death among males (HR = 0.95, 95% CI [0.92, 0.98]), and a 16% lower risk of death among females (HR = 0.84, 95% CI [0.81, 0.87]). The risk of transitioning from unaffected to non-suicide death was also significantly associated with material deprivation in adulthood; a single decile increase on the Carstairs index of deprivation was associated with a 6% higher risk of death among males (HR = 1.06, 95% CI [1.05, 1.07]) and a 4% higher risk of death among females (HR = 1.04, 95% CI [1.03, 1.06]). Among females only, a higher risk of transitioning from unaffected to non-suicide death was also significantly associated with larger family size in the mutually-adjusted model; a 1-person increase in family size was associated with a 2% higher risk of death (HR = 1.02, 95% CI [1.00, 1.04]).

Among both males and females, an increased risk of transitioning from self-harm to non-suicide death was significantly associated with lower childhood cognitive ability in the univariate models. This association remained significant in the mutually-adjusted model only among females: a 1SD advantage in childhood cognitive ability was associated with a 12% decrease in the risk of death among those living with self-harm (HR = 0.88, 95% CI [0.78, 1.00]). Among females, adulthood material deprivation and time spent in the self-harm state were also significantly associated with risk of transitioning from self-harm to non-suicide death in the mutually-adjusted model. A single decile increase in Carstairs deprivation index was associated with a 5% higher risk of death (HR = 1.05, 95% CI [1.00, 1.10]), and a single month increase in time associated with a <1% increase in risk (HR = 1.00, 95% CI [1.00, 1.01]). Among males, none of the variables – including time spent in the self-harm state – were significantly associated with risk of non-suicide death in the mutually-adjusted model.

## 4 Discussion

Using a large population cohort, the present study investigated the association between childhood cognitive ability and both self-harm and suicide across middle- and older age. Higher cognitive ability in childhood was associated with reduced risk of self-harm and non-suicide death across later-life, but not with risk of suicide (regardless of whether individuals had experienced self-harm). Meanwhile, covariates including higher material deprivation in adulthood and longer time spent living with self-harm showed consistent independent associations with higher risk of self-harm and suicide, respectively, among both males and females.

### 4.1 Self-harm

Transitions into a self-harm state were rare, particularly among males. An increased risk of self-harm was significantly associated with lower cognitive ability in childhood, and this association was similar in strength between males (HR = 0.89) and females (HR = 0.90). Early-life cognitive ability shows similar associations with mental health outcomes beyond self-harm, with lower cognitive ability associated with increased risk of depression, psychological distress and poor wellbeing, among males and females with lower cognitive ability in childhood (Bridger & Daly, 2017; Cheng & Furnham, 2013; Hatch et al., 2007; Iveson et al., 2020, 2021). However, the observed association is notably weaker than those reported in previous studies of self-harm risk. For example, in a study of Swedish conscripted males, Batty et al. reported a much stronger association between higher cognitive ability and lower risk of self-harm, even after accounting for similar covariates (HR = 0.64) (Batty et al., 2010). Note, however, that the follow-up period in Batty et al. primarily covered mid-life, where self-harm and suicide are more common than in later-life. The relative rarity of self-harm among males in the present sample – particularly when limiting the analyses to events over the age of 65 years (Supplementary Table S1) – may have limited our statistical power. At the same time, the impact of cognitive ability on self-harm risk may dissipate over time as other factors become more important, resulting in weaker associations among older adults. Indeed, the present study observed a strong association between higher levels of material deprivation in adulthood and greater risk of self-harm in later life, along with an attenuation of the association between childhood cognitive ability and self-harm risk once socioeconomic conditions were taken into account. The importance of socioeconomic deprivation for self-harm risk is consistent with work that used routinely-collected health data to track self-harm longitudinally (Carr et al., 2016; Kivimäki et al., 2020). Furthermore, hospitalisation due to self-harm has been highlighted as a starting point for the accumulation of further socioeconomically-patterned comorbidities, including liver disease, heart disease and dementia (Kivimäki et al., 2020).

### 4.2 Suicide

By using a multistate model, the present study was able to separate the suicide risk of middle-aged and older adults experiencing a self-harm event from that of adults with no recorded self-harm event during follow-up. We observed a higher risk of suicide among middle-aged and older adults experiencing self-harm, consistent with work highlighting self-harm as a major risk factor for suicide (Hawton et al., 2003; Morgan et al., 2018; Murphy et al., 2012). However, this self-harm-related increase in risk was particularly high among females. Given that suicide was more frequent among middle-aged and older males, and that self-harm events represent hospital admissions, this likely indicates less help-seeking behaviour among males (De Leo et al., 2005) rather than sex differences in self-harm per se.

Despite separate estimation of suicide risk, childhood cognitive ability was not significantly associated with transitions to suicide from either the self-harm or unaffected states. This contrasts to work examining suicide in the same sample, in which lower childhood cognitive ability was significantly associated with an increased risk of suicide among males (HR = 0.80) but not females (HR = 1.15) (Calvin et al., 2017). Note, however, that the previously observed association was not adjusted for socioeconomic circumstances such as family circumstances and material deprivation. In the present study, univariate hazard ratios were broadly similar and reached statistical significance among males, but these associations became non-significant when adjusted for socioeconomic circumstances. Given evidence of confounding by such circumstances, this suggests that, in this cohort, suicide risk may be better explained by socioeconomic conditions both in childhood and adulthood than by cognitive ability. Meanwhile, Gunnell et al. report an association between lower cognitive ability (in early adulthood) and suicide risk that was only slightly attenuated when controlling for educational attainment (Gunnell et al., 2005). While this may represent differences in the confounding contributions of education versus more material socioeconomic conditions (e.g., deprivation) or the impact of later assessment of cognitive ability, it more likely reflects fundamental differences in the frequency of suicide events and the resulting impact on statistical power. Although the rate of suicides (0.5% of all males and 0.2% of all females) is slightly higher than that observed by Gunnell et al. (0.2% of males), there are much fewer suicide events in the present study (134 males and 44 females) making it difficult to detect small but meaningful associations between cognitive ability and suicide.

Only one covariate was significantly associated with suicide risk among both males and females: time spent in the self-harm state. The risk of suicide was higher among those who spent longer in the self-harm state – i.e., among those who were further away in time from their self-harm hospitalisation. This may present a possible point for intervention to reduce suicide risk. However, it is unclear whether this represents the timing of the exposure, with earlier self-harm events posing the greatest risk, or the potential for repeated exposure, with those exposed early having the largest time window to experience self-harm again. As the present study does not account for the number of self-harm events experienced, it may be that middle-aged and older adults spending longer in the self-harm state experience repeated self-harm events, increasing the risk of suicide (Murphy et al., 2012). Further work should aim to distinguish the impact of delaying self-harm onset versus reducing repeated self-harm events.

### 4.3 Non-suicide death

Whereas suicide was a rare event, non-suicide death was relatively frequent, affecting over half of the sample over follow-up. Among unaffected individuals, lower childhood cognitive ability and higher adulthood material deprivation were both associated with increased risk of non-suicide death. Similar associations among unaffected individuals in terms of direction and strength have been have been reported both in the same sample (Calvin et al., 2017; Iveson et al., 2018; Čukić et al., 2017) and in others (Calvin et al., 2011; Christensen et al., 2016).

Non-suicide death was particularly frequent among those experiencing self-harm, affecting around 70% of individuals. This is consistent with work showing significantly reduced life expectancy among those experiencing self-harm (Bergen et al., 2012). Indeed, older adults experiencing self-harm exhibit increased risk of both suicide and non-suicide death within the year following self-harm, relative to age- and sex-matched controls without experience of self-harm (Morgan et al., 2018). Note, however, that in the present study, we observed a very weak association between longer time spent in the self-harm state and mortality risk. Among middle-aged and older females that experienced self-harm events during follow-up, an increased risk of non-suicide death was associated with lower childhood cognitive ability and higher adult deprivation. However, these associations were similar in strength to those observed in middle-aged and older females without self-harm events, with no evidence of differential contributions. None of the included factors were significantly associated with non-suicide mortality risk among middle-aged and older males experiencing self-harm, unlike among unaffected males. Indeed, the associations between mortality risk and both childhood cognitive ability and adult deprivation were weaker than those observed in females experiencing self-harm. Increased non-suicide mortality risk among middle-aged and older males experiencing self-harm may instead result from factors not captured in the present study, including greater multimorbidity and polypharmacy (Mitchell et al., 2017; Morgan et al., 2018).

### 4.4 Potential mechanisms

The observed association between higher cognitive ability and lower risk of self-harm is consistent with the notion that managing health and preventing injury are cognitively demanding processes due to the importance of health literacy, treatment adherence and problem solving (Gottfredson, 2004). A greater susceptibility to self-harm (and non-suicide death) among those with lower childhood cognitive ability may be due to indirect routes, mediated by factors arising between childhood and later-life. For example, lower childhood cognitive ability has been associated with poorer employment outcomes and quality of life (Iveson & Deary, 2019), lower adult income (Keuschnigg et al., 2023), and with poorer health behaviours (Fawns-Ritchie et al., 2018). These factors may in turn increase the risk of self-harm in later life (Rauf, 2023). Lower childhood cognitive ability may also indirectly impact self-harm risk through its contribution to insufficient coping strategies (Kitano & Lewis, 2005). The impact of lower childhood cognitive ability may also be through a more direct route. Note that this is not mutually exclusive with more indirect mechanisms. The system integrity theory (Deary, 2012) suggests that cognitive ability is an indicator of bodily functioning, something that is supported by genetic studies of cognitive ability and many health outcomes (Arden et al., 2016). For example, previous work has shown a negative genetic correlation between cognitive ability and depression (r_g_ = −0.13), indicating some shared biological basis between cognitive ability and risk of mental ill-health (Table S9; Becker et al., 2021) that may also explain variation in self-harm and suicide risk. Similarly, Gunnell et al. suggest that lower cognitive test scores in early-life may indicate impaired neurodevelopment, including in those regions of the brain implicated in psychiatric illness (Gunnell et al., 2005). For example, lower childhood cognitive ability has been associated with higher depression risk (Iveson et al., 2021; van Os, 1997), a condition that greatly increases the risk of self-harm among older adults (Chan et al., 2007; Dennis et al., 2005; Draper, 1996; Morgan et al., 2018; Szanto et al., 2002). However, psychiatric illness is unlikely to explain all of cognitive ability's association with suicide risk; cognitive ability appears to better predict self-harm risk among those without psychosis than those with psychosis (Batty et al., 2010).

### 4.5 Strengths & limitations

This study builds on previous work by extending follow-up further into later-life, up to age 85 years-old, and by modelling independent associations with suicide risk among those with and without self-harm events. Furthermore, by focussing on cognitive ability in childhood – decades before the observation of self-harm and suicide – the present study better addresses concerns over confounding. However, the present study is subject to several important limitations. Firstly, the study is limited in the range of confounders accounted for, leaving open the possibility of residual confounding. Earlier childhood socioeconomic conditions (beyond family position and size), for example, may underly some of the cognitive ability associations observed here. Additionally, factors such as geographic access to health services may have impacted the prevalence of outcomes such as self-harm admissions. There is also the potential for downstream but unobserved factors such as education, polypharmacy and comorbidity to mediate the apparent direct association between childhood cognitive ability and self-harm, suicide and death. Although these were not available in the present study, future studies should include a broad range of potential confounders and mediators to better identify the paths by which cognitive ability in childhood operates. The present study is also subject to some selection effects. The analytic sample consisted of around 75% of those children who participated in the Scottish Mental Survey 1947; individuals who could not be linked to routine health records were excluded. Notably, excluded individuals were not missing at-random. Although excluded individuals were similar to the analytic sample in several demographic measures, they exhibited higher cognitive ability, earlier family position and smaller family size than retained individuals. Such selection effects have been explored in the same cohort previously (Čukić et al., 2017), and likely indicate those most open to emigration (Bratti et al., 2020; Butikofer & Peri, 2017). These differences, particularly the small difference in childhood cognitive ability, may lead to biased survival estimates in the present study. As with many health record linkage studies, the present study does not include self-harm events that do not result in a hospital admission. This misses self-harm events that present only to primary care or emergency departments (without subsequent admission) and those that do not present to healthcare services at all, likely underestimating potential risk factors (Marchant et al., 2020). As state membership was based on presence in a given routinely-collected record (e.g., hospitalisation for self-harm), those with non-hospital presentations of self-harm will be misrepresented as transitioning straight from unaffected (entry) to suicide, death or censor. Furthermore, individuals in the main analytic sample are treated as ‘healthy’ at entry to the study; no information is available regarding the history of self-harm or existing psychiatric illness prior to the start of the study. Work has highlighted the importance of previous self-harm and previous psychiatric illness for self-harm risk in later life (Troya et al., 2019). Indeed, in the present study, sensitivity analyses of events aged 65 or over showed that having a self-harm admission prior to age 65 was strongly related to an increased risk of transitions from baseline to self-harm, suicide and non-suicide death in older age (Supplementary Tables S2 and S3). Additionally, the present study does not distinguish the intention behind self-harm events. Although all included events were deliberate, it is unclear whether they were attempts at suicide or not. Work has shown that attempted suicide makes up a large proportion of self-harm events in middle-aged and older adults (Draper, 1996; Hawton & Harriss, 2008). Self-harm events in the present sample, then, are more likely to represent suicide attempts, particularly since they require hospitalisation. Finally, the definition for self-harm and suicide events used in the present study differs to many others by only including admissions and deaths due to intentional self-harm, not those of undetermined intent. In the present study, restricting the self-harm and suicide definitions likely undercounts the number of these events in the sample and results in misclassification of individuals (King et al., 2023). However, given the rarity of self-harm and suicide events, it also likely underestimates the contribution of childhood cognitive ability. Indeed, the increased case numbers captured by a wider criteria appears to narrow confidence intervals while maintaining cognitive ability effect sizes (Calvin et al., 2017).

Perhaps most notably, the rarity of self-harm and suicide events in the present study poses a challenge in terms of the statistical power to detect meaningful associations, one that has been highlighted by several similar studies (Hawton et al., 2003; Murphy et al., 2012). This is despite the observed prevalence of suicide (0.5% of all males and 0.2% of all females) being much higher than in the general Scottish population (0.02% of males and 0.01% of females in 2021) (Scottish Public Health Observatory, 2022a). Much larger samples, such as the population-wide cohorts used in conscript studies (N = 987,308 males) (Gunnell et al., 2005), are needed to investigate these rare events. Note, however, that existing population-wide cohorts are themselves limited in their focus (e.g., males only); new longitudinal cohorts of unprecedented scale and follow-up are needed to adequately address questions about early-life risk factors for suicide.

## 5 Conclusions

Self-harm and suicide remain prevalent among middle-aged and older adults and, given the ageing population, present major challenges to public health. The present study adds to the growing literature regarding the life-long consequences of childhood disadvantage and supports the need for early-life interventions to improve health across the life course. It further demonstrates that the association between lower childhood cognitive ability and higher self-harm risk is similar between males and females, but highlights the relative rarity of self-harm events among middle-aged and particularly older adults, at least as captured by hospital admissions. It also demonstrates that the impact of lower childhood cognitive ability differs between self-harm and suicide risk. Indeed, the present study shows no significant association with suicide risk when modelled concurrently with self-harm. This suggests that tackling early-life inequalities (including in cognitive ability, through education etc.) may reduce the risk of self-harm, but may only indirectly affect suicide risk through self-harm prevention. Reducing suicide risk more specifically may be better achieved at the primary care level, through screening and management programmes (Okolie et al., 2017). Further work is required to clarify the mechanisms underlying associations with self-harm, and to re-examine such rare events in much larger cohorts.

## Ethics statement

Berkshire NHS Research Ethics Committee waived the need for full ethical review of this work. This work is instead covered under the National Safe Haven Generic Ethics approval, run by Public Health Scotland and approved by the East of Scotland NHS Research Ethics Service.

## Funding

MHI, ELB and AMM are supported by the Wellcome Trust (220857/Z/20/Z; 226770/Z/22/Z, 104036/Z/14/Z; 216767/Z/19/Z). MHI, ELB, AJ and AMM are also supported by the HDR UK DATAMIND hub, which is funded by the UKRI (MR/W014386/1). AMM is also supported by a European Union Horizon 2020 grant (agreement 847776). HCW is supported by a UKRI award (MC/PC/17209). SRC is supported by a Sir Henry Dale Fellowship jointly funded by the Wellcome Trust and the Royal Society (221890/Z/20/Z). GDB is supported by the UK Medical Research Council (MR/P023444/1) and the US National Institute on Aging (1R56AG052519-01; 1R01AG052519-01A1).

## Declarations of Interest

None.

## CRediT authorship contribution statement

**Matthew H. Iveson:** Writing – review & editing, Writing – original draft, Project administration, Methodology, Investigation, Formal analysis, Data curation, Conceptualization. **Emily L. Ball:** Writing – review & editing, Methodology, Investigation, Formal analysis, Data curation, Conceptualization. **Heather C. Whalley:** Writing – review & editing. **Ian J. Deary:** Writing – review & editing, Methodology, Conceptualization. **Simon R. Cox:** Writing – review & editing, Methodology, Funding acquisition, Conceptualization. **G. David Batty:** Writing – review & editing, Methodology. **Ann John:** Writing – review & editing. **Andrew M. McIntosh:** Writing – review & editing, Project administration, Investigation, Funding acquisition, Conceptualization.

## Data Availability

The authors do not have permission to share data.
