# Supplementary material for: Childhood cognitive ability and self-harm and suicide in later life
Source: SSM Popul Health. 2023 Dec 29;25:101592. doi: 10.1016/j.ssmph.2023.101592 (PMC10821139; doi:10.1016/j.ssmph.2023.101592)
Supplement: Multimedia component 1 [file mmc1.docx]

**Supplementary Material**

**Supplementary Figure 1.** Sample selection


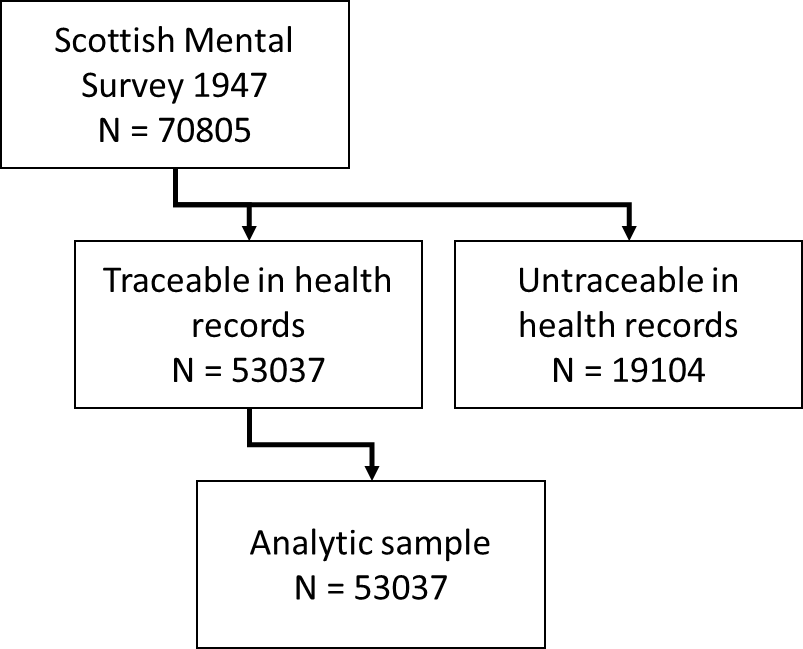


**Supplementary Figure 2.** Kaplan-Meier curves showing cumulative hazards for each transition, for males and females


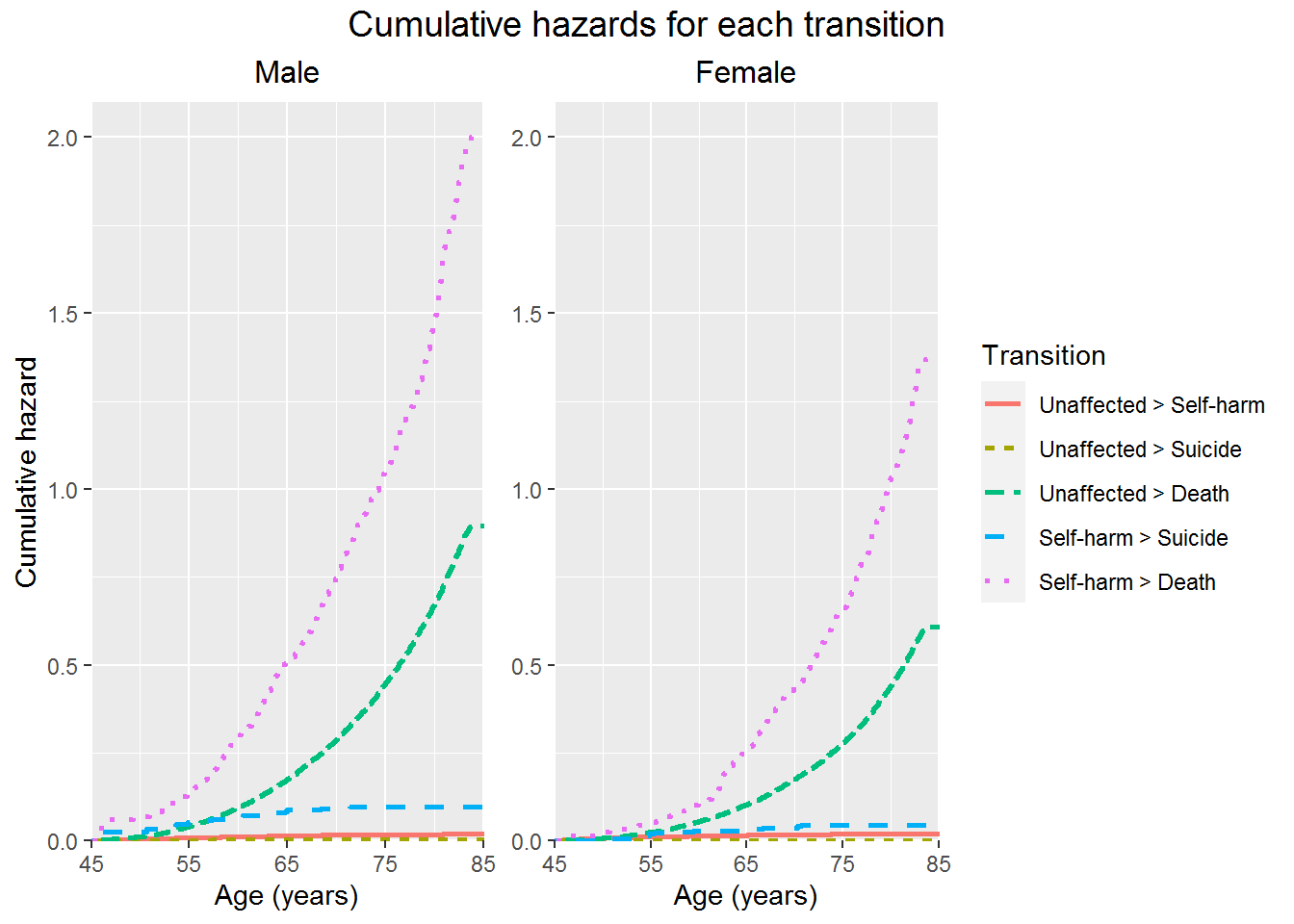


**Supplementary Figure 3.** Kaplan-Meier curves for the over-65 analysis, showing cumulative hazards for each transition, for males and females


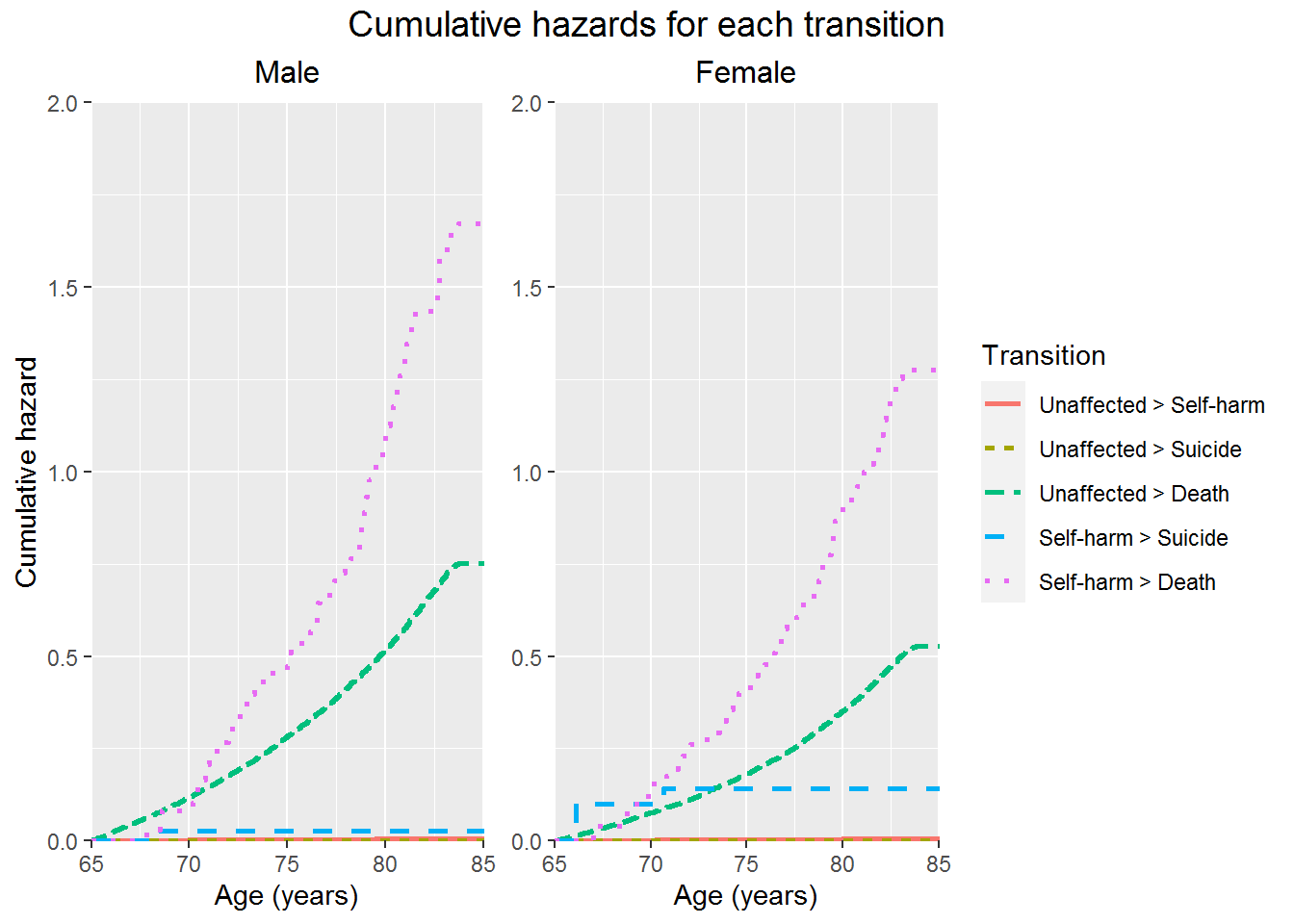


**Supplementary Table S1.** Frequency of each transitions after age 65, males and females. Diagonals represent those who stayed in each state until the end of follow-up. Infrequent transitions have been censored for disclosure control purposes.

|  | **Males**  **N = 21326** | | | | | **Females**  **N = 23451** | | | | |
| --- | --- | --- | --- | --- | --- | --- | --- | --- | --- | --- |
|  | **To** | | | | | | | | | |
| **From** |  | Unaffected | Self-harm | Suicide | Death |  | Unaffected | Self-harm | Suicide | Death |
|  | Unaffected | 9960 | 104 | <50 | 11235 | Unaffected | 13752 | 118 | <50 | 9572 |
|  | Self-harm | - | <50 | <50 | 73 | Self-harm | - | <50 | <50 | 69 |
|  | Suicide | - | - | <50 | - | Suicide | - | - | <50 | - |
|  | Death | - | - | - | 11308 | Death | - | - | - | 9641 |

**Supplementary Table S2.** Univariate and mutually-adjusted associations with each transition after the age of 65 years from the multistate model, males

|  |  | **Univariate** | | | **Mutually-adjusted** | | |
| --- | --- | --- | --- | --- | --- | --- | --- |
| **Transition**  (N transitioning/N in state) | **Variable**  (unit change) | **HR**  **[95% CI]** | **SE** | **p-value** | **HR**  **[95% CI]** | **SE** | **p-value** |
| **Unaffected > Self-harm**  (N = 104/21326) | **Childhood Cognitive ability**  (1SD higher) | 0.86  [0.71, 1.03] | 0.09 | 0.10 | 1.05  [0.86, 1.28] | 0.10 | 0.64 |
|  | **Childhood Family position**  (1 person later) | 1.11  [0.92, 1.35] | 0.10 | 0.28 | 1.18  [0.89, 1.57] | 0.15 | 0.26 |
|  | **Childhood Family size**  (1 person larger) | 1.02  [0.93, 1.11] | 0.04 | 0.72 | 0.93  [0.81, 1.06] | 0.07 | 0.25 |
|  | **Adulthood Carstairs deprivation**  (1 decile more deprived) | 1.06  [0.99, 1.14] | 0.04 | 0.08 | 1.06  [0.99, 1.14] | 0.04 | 0.09 |
|  | **Self-harm prior to age 65**  (Yes) | 34.39  [20.68, 57.52] | 0.26 | <0.001 | 9.97  [5.96, 1.67] | 0.26 | <0.001 |
| **Unaffected > Suicide**  (N = <50/21326) | **Childhood Cognitive ability**  (1SD higher) | 0.70  [0.48, 1.03] | 0.19 | 0.07 | 0.63  [0.34, 1.16] | 0.31 | 0.14 |
|  | **Childhood Family position**  (1 person later) | 1.19  [0.82, 1.74] | 0.19 | 0.36 | 1.37  [0.51, 3.68] | 0.50 | 0.53 |
|  | **Childhood Family size**  (1 person larger) | 1.06  [0.90, 1.24] | 0.08 | 0.49 | 0.68  [0.40, 1.17] | 0.28 | 0.17 |
|  | **Adulthood Carstairs deprivation**  (1 decile more deprived) | 0.89  [0.72, 1.10] | 0.11 | 0.30 | 0.87  [0.69, 1.10] | 0.12 | 0.24 |
|  | **Self-harm prior to age 65**  (Yes) | 13.48  [3.18, 57.14] | 0.74 | <0.001 | 13.35  [2.77, 6.44] | 0.80 | <0.001 |
| **Unaffected > Non-suicide death**  (N = 11235/21326) | **Childhood Cognitive ability**  (1SD higher) | 0.84  [0.82, 0.85] | 0.01 | <0.001 | 0.95  [0.92, 0.98] | 0.02 | <0.001 |
|  | **Childhood Family position**  (1 person later) | 1.10  [1.08, 1.12] | 0.01 | <0.001 | 1.04  [0.99, 1.08] | 0.02 | 0.09 |
|  | **Childhood Family size**  (1 person larger) | 1.04  [1.04. 1.05] | <0.01 | <0.001 | 1.00  [0.98, 1.02] | 0.01 | 0.95 |
|  | **Adulthood Carstairs deprivation**  (1 decile more deprived) | 1.05  [1.04, 1.07] | 0.01 | <0.001 | 1.05  [1.04, 1.06] | 0.01 | <0.001 |
|  | **Self-harm prior to age 65**  (Yes) | 2.52  [2.12, 2.96] | 0.09 | <0.001 | 1.67  [1.40, 2.00] | 0.09 | <0.001 |
| **Self-harm > Suicide**  (N = <50/104) | **Childhood Cognitive ability**  (1SD higher) | 1.42  [0.21, 9.81] | 0.99 | 0.72 | 0.52  [<0.01, 1.12e+106] | 124.89 | >0.99 |
|  | **Childhood Family position**  (1 person later) | 2.46  [0.21, 28.57] | 1.25 | 0.47 | 1207.56  [<0.01, 4.92e+170] | 196.86 | 0.97 |
|  | **Childhood Family size**  (1 person larger) | 1.15  [0.41, 3.20] | 0.52 | 0.79 | 0.03  [<0.01, 3.75e+49] | 60.04 | 0.95 |
|  | **Adulthood Carstairs deprivation**  (1 decile more deprived) | 1.73  [0.49, 6.05] | 0.64 | 0.39 | 6.88  [<0.01, 2.13e+48] | 55.78 | 0.97 |
|  | **Self-harm prior to age 65**  (Yes) | <0.01  [-0.01, 2.93e+117] | 142.32 | 0.95 | 188.47  [<0.01, >1.00e+999] | 403.37 | 0.99 |
|  | **Time in self-harm state**  (1 month longer) | - | - | - | 1.83  [<0.01, 2.88] | 8.45 | 0.94 |
| **Self-harm > Non-suicide death**  (N = 73/104) | **Childhood Cognitive ability**  (1SD higher) | 0.79  [0.64, 0.99] | 0.11 | 0.04 | 0.87  [0.68, 1.11] | 0.12 | 0.28 |
|  | **Childhood Family position**  (1 person later) | 1.28  [1.02, 1.62] | 0.12 | 0.04 | 1.10  [0.79, 1.53] | 0.17 | 0.56 |
|  | **Childhood Family size**  (1 person larger) | 1.14  [1.04, 1.26] | 0.05 | 0.01 | 1.06  [0.91, 1.23] | 0.08 | 0.44 |
|  | **Adulthood Carstairs deprivation**  (1 decile more deprived) | 1.10  [1.01, 1.20] | 0.04 | 0.03 | 1.06  [0.97, 1.17] | 0.05 | 0.19 |
|  | **Self-harm prior to age 65**  (Yes) | 1.21  [0.68, 2.15] | 0.29 | 0.51 | 1.12  [0.62, 2.02] | 0.30 | 0.70 |
|  | **Time in self-harm state**  (1 month longer) | - | - | - | 1.01  [1.00, 1.01] | <0.01 | 0.03 |

**Supplementary Table S3.** Univariate and mutually-adjusted associations with each transition after the age of 65 years from the multistate model, females

|  |  | **Univariate** | | | **Mutually-adjusted** | | |
| --- | --- | --- | --- | --- | --- | --- | --- |
| **Transition**  (N transitioning/N in state) | **Variable**  (unit change) | **HR**  **[95% CI]** | **SE** | **p-value** | **HR**  **[95% CI]** | **SE** | **p-value** |
| **Unaffected > Self-harm**  (N = 118/23451) | **Childhood Cognitive ability**  (1SD higher) | 0.87  [0.72, 1.06] | 0.10 | 0.16 | 1.04  [0.84, 1.29] | 0.11 | 0.70 |
|  | **Childhood Family position**  (1 person later) | 1.19  [1.00, 1.43] | 0.09 | 0.05 | 1.17  [0.89, 1.54] | 0.14 | 0.27 |
|  | **Childhood Family size**  (1 person larger) | 1.06  [0.99, 1.15] | 0.04 | 0.11 | 1.00  [0.88, 1.12] | 0.06 | 0.95 |
|  | **Adulthood Carstairs deprivation**  (1 decile more deprived) | 1.01  [0.94, 1.07] | 0.03 | 0.84 | 0.99  [0.92, 1.06] | 0.03 | 0.69 |
|  | **Self-harm prior to age 65**  (Yes) | 30.71  [19.59, 48.16] | 0.23 | <0.001 | 10.46  [6.58, 16.64] | 0.24 | <0.001 |
| **Unaffected > Suicide**  (N = <50/23451) | **Childhood Cognitive ability**  (1SD higher) | 1.99  [0.83, 4.74] | 0.44 | 0.12 | 1.61  [0.54, 4.80] | 0.56 | 0.39 |
|  | **Childhood Family position**  (1 person later) | 0.89  [0.46, 1.73] | 0.34 | 0.74 | 1.23  [0.33, 4.64] | 0.68 | 0.76 |
|  | **Childhood Family size**  (1 person larger) | 0.87  [0.62, 1.23] | 0.18 | 0.44 | 0.89  [0.46, 1.72] | 0.34 | 0.73 |
|  | **Adulthood Carstairs deprivation**  (1 decile more deprived) | 0.85  [0.63, 1.14] | 0.15 | 0.27 | 0.95  [0.69, 1.31] | 0.16 | 0.69 |
|  | **Self-harm prior to age 65**  (Yes) | 14.18  [1.77, 113.80] | 1.06 | 0.01 | 11.28  [1.23, 103.07] | 1.13 | 0.03 |
| **Unaffected > Non-suicide death**  (N = 9572/23451) | **Childhood Cognitive ability**  (1SD higher) | 0.79  [0.77, 0.81] | 0.01 | <0.001 | 0.85  [0.82, 0.88] | 0.02 | <0.001 |
|  | **Childhood Family position**  (1 person later) | 1.08  [1.06, 1.10] | 0.01 | <0.001 | 0.97  [0.93, 1.01] | 0.02 | 0.16 |
|  | **Childhood Family size**  (1 person larger) | 1.04  [1.03, 1.05] | <0.01 | <0.001 | 1.02  [1.00, 1.04] | 0.01 | 0.03 |
|  | **Adulthood Carstairs deprivation**  (1 decile more deprived) | 1.06  [1.05, 1.07] | 0.01 | <0.001 | 1.04  [1.03, 1.05] | 0.01 | <0.001 |
|  | **Self-harm prior to age 65**  (Yes) | 2.43  [2.08, 2.84] | 0.08 | <0.001 | 1.60  [1.35, 1.89] | 0.09 | <0.001 |
| **Self-harm > Suicide**  (N = <50/118) | **Childhood Cognitive ability**  (1SD higher) | 0.35  [0.10, 1.17] | 0.62 | 0.09 | 0.01  [<0.01, 6.77e+71] | 86.87 | 0.95 |
|  | **Childhood Family position**  (1 person later) | 58.53  [0.77, 4472.37] | 2.21 | 0.07 | 0.03  [<0.01, 2.08e+104] | 124.26 | 0.98 |
|  | **Childhood Family size**  (1 person larger) | 3.33  [0.92, 12.01] | 0.66 | 0.07 | 47.51  [<0.01, 3.50e+55] | 63.26 | 0.95 |
|  | **Adulthood Carstairs deprivation**  (1 decile more deprived) | 0.99  [0.63, 1.54] | 0.23 | 0.95 | 1.32  [<0.01, 3.96e+27] | 32.27 | 0.99 |
|  | **Self-harm prior to age 65**  (Yes) | <0.01  [<0.01, 7.40e+157] | 190.89 | 0.96 | 210.37  [<0.01, 5.60e+281 | 328.18 | 0.99 |
|  | **Time in self-harm state**  (1 month longer) | - | - | - | 1.58  [<0.01, 1.00e+999] | <0.01 | 0.99 |
| **Self-harm > Non-suicide death**  (N = 69/118) | **Childhood Cognitive ability**  (1SD higher) | 0.93  [0.73, 1.20] | 0.13 | 0.60 | 1.00  [0.76, 1.31] | 0.14 | 0.98 |
|  | **Childhood Family position**  (1 person later) | 1.25  [0.98, 1.60] | 0.12 | 0.07 | 1.07  [0.74, 1.54] | 0.19 | 0.71 |
|  | **Childhood Family size**  (1 person larger) | 1.09  [0.98, 1.21] | 0.06 | 0.12 | 1.08  [0.91, 1.27] | 0.08 | 0.38 |
|  | **Adulthood Carstairs deprivation**  (1 decile more deprived) | 1.03  [0.94, 1.12] | 0.04 | 0.50 | 1.04  [0.95, 1.15] | 0.05 | 0.39 |
|  | **Self-harm prior to age 65**  (Yes) | 0.98  [0.56, 1.72] | 0.29 | 0.95 | 0.93  [0.51, 1.68] | 0.30 | 0.81 |
|  | **Time in self-harm state**  (1 month longer) | - | - | - | 1.00  [0.99, 1.01] | <0.01 | 0.78 |

**Supplementary Table S4.** Mutually-adjusted associations with each transition from the multistate model, males, without adult socioeconomic conditions

| **Transition**  (N transitioning/N in state) | **Variable**  (unit change) | **HR**  **[95% CI]** | **SE** | **p-value** |
| --- | --- | --- | --- | --- |
| **Unaffected > Self-harm**  (N = 451/26234) | **Childhood Cognitive ability**  (1SD higher) | 0.77  [0.70, 0.85] | 0.05 | <0.001 |
|  | **Childhood Family position**  (1 person later) | 1.03  [0.90, 1.19] | 0.07 | 0.63 |
|  | **Childhood Family size**  (1 person larger) | 1.01  [0.95, 1.07] | 0.03 | 0.76 |
| **Unaffected > Suicide**  (N = 118/26234) | **Childhood Cognitive ability**  (1SD higher) | 0.87  [0.72, 1.05] | 0.10 | 0.14 |
|  | **Childhood Family position**  (1 person later) | 1.03  [0.79, 1.34] | 0.14 | 0.83 |
|  | **Childhood Family size**  (1 person larger) | 1.06  [0.94, 1.19] | 0.06 | 0.35 |
| **Unaffected > Non-suicide death**  (N = 15230/26234) | **Childhood Cognitive ability**  (1SD higher) | 0.85  [0.84, 0.87] | 0.01 | <0.001 |
|  | **Childhood Family position**  (1 person later) | 1.05  [1.03, 1.08] | 0.01 | <0.001 |
|  | **Childhood Family size**  (1 person larger) | 1.01  [0.99, 1.02] | 0.01 | 0.32 |
| **Self-harm > Suicide**  (N = 16/451) | **Childhood Cognitive ability**  (1SD higher) | 1.09  [0.64, 1.86] | 0.27 | 0.76 |
|  | **Childhood Family position**  (1 person later) | 2.98  [1.16, 7.61] | 0.48 | 0.02 |
|  | **Childhood Family size**  (1 person larger) | 0.62  [0.40, 0.98] | 0.23 | 0.04 |
|  | **Time in self-harm state**  (1 month longer) | 1.01  [1.00, 1.02] | 0.01 | 0.02 |
| **Self-harm > Non-suicide death**  (N = 349/451) | **Childhood Cognitive ability**  (1SD higher) | 0.90  [0.80, 1.01] | 0.06 | 0.07 |
|  | **Childhood Family position**  (1 person later) | 0.90  [0.77, 1.05] | 0.08 | 0.18 |
|  | **Childhood Family size**  (1 person larger) | 1.06  [0.99, 1.13] | 0.04 | 0.12 |
|  | **Time in self-harm state**  (1 month longer) | 1.00  [1.00, 1.00] | <0.01 | 0.15 |

**Supplementary Table S5.** Mutually-adjusted associations with each transition from the multistate model, females, without adult socioeconomic conditions

| **Transition**  (N transitioning/N in state) | **Variable**  (unit change) | **HR**  **[95% CI]** | **SE** | **p-value** |
| --- | --- | --- | --- | --- |
| **Unaffected > Self-harm**  (N = 516/26803) | **Childhood Cognitive ability**  (1SD higher) | 0.76  [0.69, 0.84] | 0.05 | <0.001 |
|  | **Childhood Family position**  (1 person later) | 1.02  [0.90, 1.16] | 0.06 | 0.73 |
|  | **Childhood Family size**  (1 person larger) | 1.02  [0.96, 1.08] | 0.03 | 0.49 |
| **Unaffected > Suicide**  (N = 31/26803) | **Childhood Cognitive ability**  (1SD higher) | 1.30  [0.84, 2.02] | 0.22 | 0.24 |
|  | **Childhood Family position**  (1 person later) | 1.30  [0.74, 2.30] | 0.29 | 0.36 |
|  | **Childhood Family size**  (1 person larger) | 0.81  [0.60, 1.10] | 0.15 | 0.17 |
| **Unaffected > Non-suicide death**  (N = 11990/26803) | **Childhood Cognitive ability**  (1SD higher) | 0.80  [0.78, 0.81] | 0.01 | <0.001 |
|  | **Childhood Family position**  (1 person later) | 1.02  [1.00, 1.05] | 0.01 | 0.08 |
|  | **Childhood Family size**  (1 person larger) | 1.01  [0.99, 1.02] | 0.01 | 0.37 |
| **Self-harm > Suicide**  (N = 13/516) | **Childhood Cognitive ability**  (1SD higher) | 1.21  [0.63, 2.32] | 0.33 | 0.57 |
|  | **Childhood Family position**  (1 person later) | 0.65  [0.30, 1.42] | 0.40 | 0.28 |
|  | **Childhood Family size**  (1 person larger) | 1.29  [0.91, 1.85] | 0.18 | 0.16 |
|  | **Time in self-harm state**  (1 month longer) | 1.04  [1.02, 1.06] | 0.01 | <0.001 |
| **Self-harm > Non-suicide death**  (N = 350/516) | **Childhood Cognitive ability**  (1SD higher) | 0.86  [0.76, 0.97] | 0.06 | 0.01 |
|  | **Childhood Family position**  (1 person later) | 1.01  [0.86, 1.19] | 0.08 | 0.87 |
|  | **Childhood Family size**  (1 person larger) | 1.01  [0.94, 1.09] | 0.04 | 0.73 |
|  | **Time in self-harm state**  (1 month longer) | 1.00  [1.00, 1.01] | <0.01 | <0.001 |
